# Effects of Blood-Derived Products on Cellular Senescence and Inflammatory Response: A Study on Skin Rejuvenation

**DOI:** 10.3390/cimb46030122

**Published:** 2024-02-28

**Authors:** Harald Kühnel, Markus Pasztorek, Olga Kuten-Pella, Karina Kramer, Christoph Bauer, Zsombor Lacza, Stefan Nehrer

**Affiliations:** 1 Center for Regenerative Medicine, University for Continuing Education Krems, Dr.-Karl-Dorrek-Straße 30, 3500 Krems an der Donau, Austria; olga.kuten@orthosera.com (O.K.-P.); karina.kramer@donau-uni.ac.at (K.K.); christoph.bauer@donau-uni.ac.at (C.B.); stefan.nehrer@donau-uni.ac.at (S.N.); 2 Department of Applied Life Science, Bioengineering, FH-Campus Vienna, Favoritenstrasse 222, 1100 Vienna, Austria; 3 Center for Experimental Medicine, University for Continuing Education Krems, Dr.-Karl-Dorrek-Straße 30, 3500 Krems an der Donau, Austria; markus@pregenerate.net; 4 Orthosera GmbH, 3500 Krems an der Donau, Austria; zlacza@mac.com; 5 Institute of Clinical Experimental Research, Semmelweis University, 1094 Budapest, Hungary; 6 Institution of Sport and Health Sciences, University of Physical Education, 1123 Budapest, Hungary

**Keywords:** blood derived products, cellular senescence, HDF, skin

## Abstract

Blood-derived products, such as citrate platelet-rich plasma (CPRP) and hyperacute serum (HAS), are recognized for their rich growth factor content. When human dermal fibroblast (HDF) cells are exposed to combined mitogenic and DNA-damaging stimuli, it can lead to an increased burden of senescent cells and a modified senescence-associated secretory phenotype. In this study, the senescent state was comprehensively assessed through various methods, including phosphorylated histone H2AX (γH2AX) staining, p21 and p16 q-PCR, p21-western blot, growth curves, and senescence-associated ß-galactosidase staining. Two primary treatments with blood products were administered, one early (immediately after etoposide) and the other late (11 days after etoposide treatment). The effects of the blood product treatment were evaluated by measuring interleukin 6 and 8 (IL-6 and IL-8) levels, as well as collagen 1 (COL1) and p21 mRNA expression. Additionally, 2,3-bis-(2-methoxy-4-nitro-5-sulfophenyl)-2H-tetrazolium-5-carboxanilide (XTT) assays, cell size measurements, viability assays, and cell number calculations were conducted. The results revealed that cells treated with hyperacute serum in the early treatment phase exhibited the lowest observed IL-6 and IL-8 levels. In contrast, a clear inflammatory response for IL-8 was observed in cells treated with hyperacute serum and citrate platelet-rich plasma during the late treatment. Furthermore, an upregulation of COL1 expression was observed in the early treatment, while cells in the late treatment group remained unaffected. Notably, citrate platelet-rich plasma-treated cells showed a decrease in COL1 expression. Overall, the treatment with blood products appears to have slightly positive effects on skin rejuvenation.

## 1. Introduction

Cellular senescence has emerged as a significant contributor to age-related degenerative diseases [1,2]. This phenomenon, characterized by the irreversible growth arrest of human diploid cells, is a defense mechanism against unlimited cellular proliferation that was first described by Leonard Hayflick and Paul Moorhead [3]. While initially linked to telomere attrition, cellular senescence can also be induced by activated oncogenes or DNA damage (stress-induced premature senescence (SIPS [4])) [5]. Unlike apoptotic cells, senescent cells remain metabolically active and accumulate with age. Furthermore, senescent cells are involved in fundamental physiological processes like wound healing [6].

Cellular senescence is a complex process with dual implications. On the one hand, it serves as a safeguard against cancer, while on the other, it activates the immune system through the establishment of the senescence-associated secretory phenotype (SASP) [7]. 

This phenotype involves the secretion of inflammatory factors into the surrounding tissue, which can have far-reaching effects on various physiological processes. The interplay between the SASP, cancer, and cellular senescence is intricate, as the factors of the SASP can attract immune cells such as M1-type macrophages, which play a crucial role in preventing the transformation of epithelial cells into carcinoma [8]. However, it is important to note that, while inflammation is essential for processes like wound healing and protection against infections, prolonged and persistent inflammation can be detrimental [9].

Specific factors of the SASP, such as IL-6 and IL-8, are of particular interest due to their involvement in inflammatory responses, age-related pathologies, and cancer. The inhibition of the SASP is being considered as an alternative to senolytic therapy to mitigate the adverse effects of senescent cells, while the activation of the immune system to remove these cells is also being explored as a potential strategy [10]. Furthermore, the secretion of inflammatory cytokines by the SASP can contribute to systemic inflammation in older organisms and potentially promote malignant transformation. It is evident that the regulation of the SASP and its impact on various physiological and pathological processes, including cancer, is a complex and multifaceted area of study that requires further investigation [11].

The SASP is triggered by severe or irreparable DNA damage, leading to the induction of senescence in normal cells. This process is initiated by the activation of Ataxia Telangiectasia Mutated (ATM), which, in turn, activates the histone H2AX by phosphorylation (γH2AX), resulting in the formation of characteristic foci. Subsequently, the phosphorylation of p53 at serine 15 upregulates p21, a cell cycle inhibitor [12]. If the DNA damage, particularly the double-strand breaks, cannot be repaired, constitutive DNA double-strand repair (DDR) signaling and p53-dependent growth arrest can lead to irreversible senescence [10,13]

Etoposide is a chemotherapeutic agent that has been used for over 20 years to treat a wide range of human cancers. It is one of the most commonly prescribed anticancer drugs worldwide, with topoisomerase II as its target. This ubiquitous enzyme causes transient double-strand breaks in the double helix during the repair processes that it catalyzes [14,15]. In the context of research on cellular senescence, etoposide-induced senescence is associated with p53 activation in human dermal fibroblasts (HDFs) [16]. Consequently, etoposide-induced senescence is an optimal model for studying the senescence-associated secretory phenotype (SASP) in HDF cells [17]

Skin rejuvenation is an important area of research due to the undesirable aesthetic effects and weakening of the skin with age. The maintenance of skin health and beauty is widely regarded as a key indicator of overall health and well-being. Consequently, there has been a rapid increase in the demand for skin rejuvenation strategies, with the global annual expenditure projected to rise from USD 24.6 billion to approximately USD 44.5 billion by 2030 [18]. However, skin aging is not just a cosmetic issue, as it can lead to various dermatological disorders that significantly impact the quality of life of older individuals. These disorders range from pruritus to more serious conditions such as melanomas [19].

The main components of the dermal stroma are HDFs and the surrounding matrix. This post-mitotic tissue is susceptible to the accumulation of dysfunctional cells over time [20,21]. Investigating HDF senescence is a crucial task in studying skin aging, given the evidence for the SASP and “SASP-like” inflammation driving skin carcinogenesis. This emphasizes how a further understanding of both the roles and mechanisms of SASP expression may offer new targets for skin cancer prevention and therapy [22].

The utilization of blood-derived products is increasingly significant in the fields of regenerative medicine and plastic surgery, particularly in the context of aging skin [23]. Numerous clinical studies have been conducted to investigate their relevance to skin aging [24]. Within the literature, various types of blood-derived products have been described, such as platelet-rich plasma (PRP) and hyperacute serum (hypACT or HAS) [25].

PRP is the most prominent type of blood product that has already been tested in skin photoaging [26]. In general, these products are known to have a high content of growth factors. HAS has more pronounced effects on proliferation than PRP in human mesenchymal stem cells. Additionally, the upregulation of BCL2 mRNA was observed after treatment with 10% HAS [27]. Furthermore, HAS stimulates growth in chondrocytes [28]. Several growth factors have also been shown to have positive effects on skin rejuvenation [29,30] by the stimulation of HDF proliferation. The rejuvenation strategy is based on the overgrowth of damaged and old cells by the proliferation of the young and healthy HDFs. Nevertheless, old cells remain in the tissue and can produce SASP-Factors. 

This study will focus on the complex effects of blood-derived products on fibroblasts, aiming to shed light on the interplay between the growth activation of young cells and the secretion of inflammatory factors from nascent and established cellular senescent cells.

## 2. Materials and Methods

### 2.1. Cell Lines and Media

HDF (human dermal fibroblast) cells were obtained from cell Research Corp (Tebu-Bio product code NF170); they were extracted from the wrist skin of a 51-year-old male and were grown in growth medium (GIBCO DMEM/F12 GlutaMAX-I; Invitrogen, LifeTech Austria, Vienna, Austria) containing antibiotics (penicillin 100 U/mL; streptomycin 0.1 mg/mL) and amphotericin B (2.5 μg/mL) (Sigma-Aldrich Chemie GmbH, Steinheim, Germany) (the fungicide was used because we were using the equipment for primary culture to prevent contamination) supplemented with 10% FCS (fetal calf serum) [GIBCO, Invitrogen, LifeTech Austria]. This growth medium was used as positive control condition in all blood product experiments and stated as “FCS” condition in all experiments. In general, this medium was used unless otherwise described. 

Cells were seeded in a density of 3500 cells/cm^2^ for all experiments [31]. Low seeding densities are important for the development of senescent state. Cells must not be contact-inhibited.

Viability was determined via trypan blue (Sigma-Aldrich Chemie GmbH) dye exclusion and cells were counted using LUNA cell counter (logos biosystems, Anyang, Republic of Korea). This is a chamber-based system using trypan blue exclusion for counting dead cells. Cells were passaged every 3 to 4 days.

### 2.2. Preparation of Blood Products

hypACT was prepared as described in Kardos et al. [25]. Briefly, whole blood was drawn from 8 male and female healthy donors (25–45 years) with the hypACT device (catalogue number: 700194, OrthoSera, Krems an der Donau, Austria) according to the manufacturer’s protocol. The sample was immediately centrifuged at 1710× *g* for 5 min at room temperature. After centrifugation, a top layer (platelet-rich fibrin clot) and a bottom layer were formed. The bottom layer, primarily containing red blood cells, was removed. 

Whole blood from the same 8 donors (women and men) as in the case of hypACT preparation was drawn into VACUETTE 9 mL 9NC Trinatriumcitrat 3.2% blood collection tubes (catalogue number: 455322; Greiner Bio-one, Kremsmünster, Austria) and centrifuged at 440× *g* for 10 min at room temperature. Three layers were formed in the tube: (i) a bottom layer (red blood cells), (ii) middle layer (with the buffy coat), and (iii) top layer (plasma). Leukocyte-poor PRP was obtained by top/plasma layer aspiration without the middle layer (buffy coat). The aspirate was transferred into a 15 mL falcon tube and centrifuged again at 1700× *g* for 10 min. The formed platelet pellet was resuspended in 50% of the remaining platelet-poor plasma supernatant. Blood-derived products were prepared following the method of Neubauer et al. [32].

Blood-derived products were pooled and either immediately used for experiments or stored at −80 °C for later use. Due to the use of citrate as an anticoagulant, the final PRP product was called CPRP to comply with the nomenclature used in our previous publications [27,32,33].

### 2.3. Growth Curves

Growth curves were generated by serial passaging of cells. Cells were seeded 1:3 or 1:6 and passaged when they reached confluence. The control curve was generated until population doubling (PD) above 40, just to show that replicative potential was not exhausted in PD when experiments were carried out.

### 2.4. Procedure for SA-ß-gal Staining

**Figure 1:**
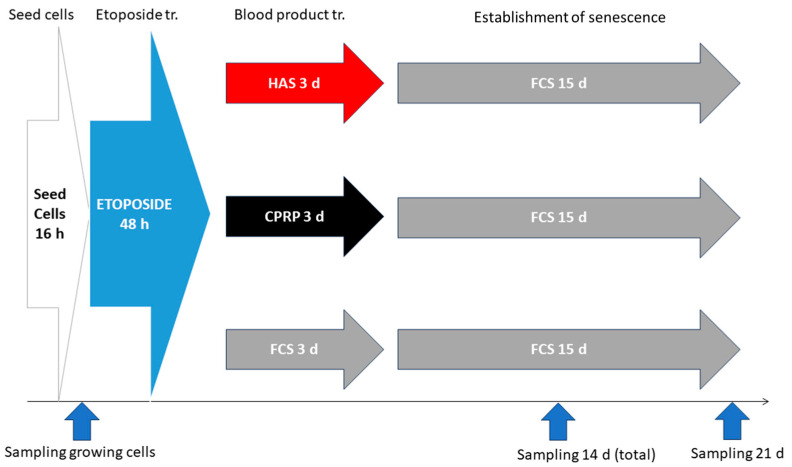
Early treatment scheme: cells were seeded 16 h (appr. 1 d) before etoposide exposure for 48 h (2 d), then blood products CPRP and HAS were applied for 3 d incubation, while normal growth medium containing FCS served as positive control. A final incubation with growth medium for 15 d completed a total of 21 days of treatment. Samples were taken on days 14 and 21. Freshly seeded cells were used as growing cell control.

**Figure 2:**
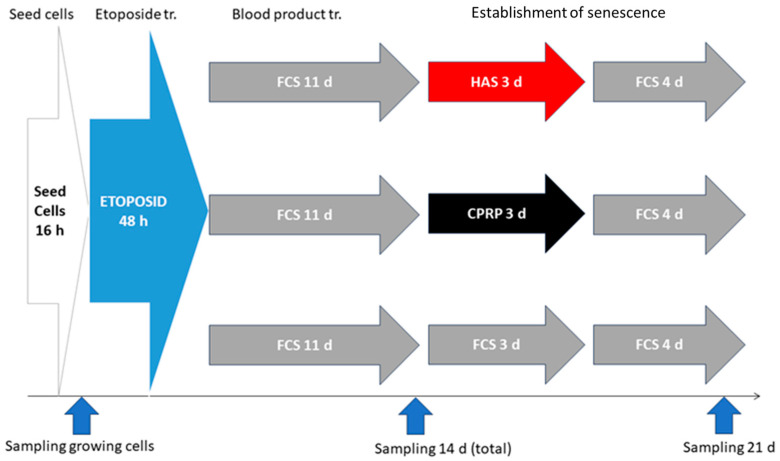
Late treatment scheme: seeding and etoposide treatments equivalent to the early treatment were followed by 11 d of incubation with growth medium (FCS) for the establishment of cellular senescence. In the next step, 3 d of blood product treatment (HAS, CPRP, and FCS) was performed, followed by 4 d of incubation in FCS medium. Sampling was carried out on days 14 and 21. Freshly seeded cells were used as growing cell control.

Cells were treated as in Figure 1 SA-ß-gal. Staining was performed at the 14-day sample point. This timepoint was chosen to confirm establishment of cellular senescence before late treatment with blood products (Figure 2). An SA-ß-gal assay was performed following the protocol of Dimri et al. [34]. Briefly, cell culture medium was aspirated, and cells were washed with PBS twice. After the last rinse, cells were incubated for fixation in PBS containing 4% formaldehyde at room temperature for 5 min. The fixation solution was aspirated, and cells were washed twice with PBS for 5 min at room temperature with gentle shaking. SA-β-gal staining solution was added (containing 0.1% X-gal, 5 mM potassium ferrocyanide, 5 mM potassium ferricyanide, 150 mM Sodium chloride, and 2 mM magnesium chloride in 40 mM citric acid/sodium phosphate solution, pH 6.0). Cells were incubated in the dark in a 37 °C incubator without CO_2_. 

### 2.5. Immuno Fluorescence

After seeding in chamber slides for 16 h, cells were incubated in 50 µM etoposide for 2 d. Following etoposide exposure, blood-derived products were added for 1 day of incubation (FCS, CPRP, and HAS at 10% each). Control cells were incubated in normal growth medium without damaging treatment. Briefly, cells were fixed in 4% formaldehyde in PBS for 15 min at room temperature. The fixative was aspirated, and cells were rinsed three times in PBS for 5 min. Blocking was performed for 60 min with PBS supplemented with 5% normal serum (FCS) and 0.3% Triton™ X-100. After aspiration of the blocking agent, the primary antibody Phospho-Histone H2A.X (Ser139) Antibody (#2577 cell signaling) was added for overnight incubation at 4 °C. After rinsing three times in PBS for 5 min, specimens were incubated in fluorochrome-conjugated secondary antibodyI, goat-anti-rabbit polyclonal Fab fragment Ab labeled with AF-488 (3 µg/mL, Jackson Laboratories, Bar Harbor, MN, USA), diluted in antibody dilution buffer (1% BSA in PBS, 0.3% Triton™ X-100) for 1 h at room temperature in dark, and then rinsed in PBS as above. Nuclei were stained with DAPI Sigma Aldrich (D9542) 1:1000 in antibody dilution buffer for 10 min at 37 °C. Slides were washed with PBS and mounting was achieved by adding 20 µL Fluoromount-GTM (Southern Biotechnology, Birmingham, AL, USA; Thermo Fisher ScientificWaltham, MA, USA). Slides were covered with high-precision coverslip No. 1.5H.

### 2.6. Western Blot Analysis

For Western blot analysis, cells were lysed in 1× Cell Lysis Buffer #9803 (Cell Signaling, Danvers, Massachusetts, MA, USA) and protein content was determined by DC assay (Biorad, Hercules, CA, USA). Equal amounts of Total Protein were separated by electrophoresis. Separated proteins were blotted to a 0.2 m PVDF membrane (Biorad) with a trans blot turbo system (Biorad). The blot was blocked with 4% dry milk in TBS-Tween 20 (0.1%) for one hour and 1:1000 p21 Waf1/Cip1 (12D1) Rabbit mAb #2947 (Cell Signaling) in blocking buffer was added consecutively and incubated over night at 4 °C. Goat anti-Rabbit IgG (H + L) Secondary Antibody, HRP (Thermo Fisher scientific) was added after washing 3 times with TBS-Tween 20 (0.1%) for 1 h and detected with Novex™ HRP Chromogenic Substrate (TMB, Kennett Square, PA, USA). Images were taken with ChemiCoc imaging system (Biorad).

### 2.7. XTT Assay

HDF were seeded in standard growth medium in a 96-well plate (3500 cells/cm^2^). The CPRP-supplemented medium contained 2 U/mL heparin (Clexane, Sanofi Aventis, Paris, France) to prevent clotting in the culture medium. Cell-free wells were used as a technical background. Cell viability was determined using Cell Proliferation Kit II (XTT; Roche, Mannheim, Germany) according to the manufacturer’s instructions at different time points. Absorbance was measured after 4 h of incubation in the staining solution using a PowerWave microplate spectrophotometer (BioTek, Winooski, VT, USA) at 480 nm with a reference wavelength at 650 nm. Relevant experiments were performed in duplicates or triplicates.

Due to the generally high time requirements for individual experiments, only two independent outgrowth experiments were conducted in triplicates.

### 2.8. BP-Treatment for Early Treatemnt Group

Three independent experiments were conducted.

#### 2.8.1. Cell Seeding

For every approach, one vial of cells was thawed at passage 7. Cells were expanded for one or two passages (max passage no. 10). Supernatants were collected at each media change. Cells were seeded at a density of 3500 cells/cm^2^ in T75 culture flasks and 15 mL medium was added. 

#### 2.8.2. Etoposide Treatment 

After adherence of cells for at least 16 h, cells were treated to 15 mL of medium, to which 15 µL of etoposide in DMSO (50 mM) was added (to a final concentration of 50 µM), and cells were incubated for 2 d. After 2 d, the medium was exchanged and the etoposide was removed.

#### 2.8.3. BP-Treatment

For treatment 1, 10% HAS (1.5 mL HAS + 13.5 mL media w/o FCS) was added, for treatment 2 10%, CPRP (1.5 mL CPRP + 3 µL Heparin + 13.5 mL media w/o FCS), and for treatment 3 10%, FCS (the latter preparation was also used for the late exposure to BP experiment). Cells were exposed to BP for three days.

#### 2.8.4. Establishment of Cellular Senescence

After the BP pulse, cells were incubated in normal growth media with medium exchange every 4 days. On day 8 after BP-treatment, three flasks were harvested. 

#### 2.8.5. Harvesting of Cells

One flask of each treatment was harvested by accutase treatment (750 µL). The reaction was stopped by adding 4.25 mL of growth medium. Cells were centrifuged at 500× *g* for 10 min and resuspended in 250 µL medium. Two aliquots of 100 µL of this suspension were centrifuged again (500× *g* and 10 min). One pellet was lysed in 250 µL RIPA buffer (+1:100 Protease inhibitor). The other pellet was stored at −80 °C for RNA extraction. A total of 20 µL of the remaining suspension was used for counting with a LUNA cell counter.

The same sampling procedure was repeated on day 14 (total treatment time) and day 21 at the end of the experiment. Additionally, the discarded medium at every medium exchange was collected and stored at −80 °C for Luminex analysis 

### 2.9. BP-Treatment for Late Exposure Group

Three independent experiments were conducted according to the treatment schedule outlined in Figure 2.

#### 2.9.1. Seeding of Cells and Etoposide Treatment

These steps were performed in the same way as in the early exposure group.

#### 2.9.2. Establishment of Cellular Senescence

After etoposide treatment, cells were incubated in normal growth medium for 11 days with medium exchange every 4 days.

#### 2.9.3. BP-Treatment

The BP-treatment was performed the same way as in the early exposure group, but with cells with established cellular senescence. 

#### 2.9.4. Final Incubation and Harvest

After a BP pulse for 3 days, cells were incubated for another 4 days and then harvested as described in Section 2.8.5. 

### 2.10. RNA Extraction and qPCR

RNA extraction: For each treatment, cells were collected and pooled groupwise in 200 µL PBS. RNA was isolated using the High Pure RNA Isolation kit (catalogue number: 11828665001, Roche Diagnostics GmbH, Mannheim, Germany) according to the manufacturer’s protocol. RNA was eluted and stored at −80 °C until cDNA synthesis. 

Gene Expression Analysis: cDNA-Synthesis was performed using Transcriptor First Strand cDNA Synthesis Kit (catalogue number: 04379012001, Roche, Basel, Switzerland). 

RT-qPCR was performed using FastStart Essential DNA Probes Master kit (catalogue number: 06402682001, Roche, Basel, Switzerland) in triplicates using the LightCycler^®^ 96 from Roche. Then, 1 µL of the cDNA product, FastStart Probe Master 2X, hydrolysis probe (final concentration 250 nM), and primers (final concentration 900 nM) were used for PCR amplification. Primers for each gene were designed to span introns to exclude genomic contamination in PCR products, using the Universal ProbeLibrary System Assay Design. GAPDH was used as a housekeeping gene. PCR conditions were optimized for annealing temperature and limited cycle number to ensure that product formation was in the responsive range.

GAPDH: (left: 5′ CTCTGCTCCTCCTGT 3′ right: 5′ ACGACCAAATCCGTT 3′ universal primer library #60) 

COL1: (left: 5′ GGGATTCCCTGGACC 3′ right: 5′ GGAACACCTCGCTCT 3′ universal primer library #67) 

p21: (left: 5′ TCACTGTCTTGTACCCTTGTGC 3′ right: 5′ GGC GTT TGG AGT GGT AGA AA 3′ universal primer library #32)

### 2.11. Luminex Assay

Supernatants (15 mL) were harvested from treated samples at the previously indicated time points and stored at −80 °C. Cytokines and chemokines, including tumor necrosis factor-alpha (TNFα), monocyte chemoattractant protein 1 (MCP1), Interferon-gamma (IFNγ), Interleukin 10 (IL-10), Interleukin 8 (IL-8), Interleukin 6 (IL-6), interleukin 1ß (IL-1ß), and interleukin-1 receptor antagonist (IL1RA), were measured with Bio-Plex Pro Assays (BIO-RAD Laboratories, Inc., Hercules, CA, USA). Analyses were performed strictly following the instructions of the supplier.

### 2.12. Statistics

Normality testing was performed using the Shapiro–Wilk test. Differences between groups were determined by simple ANOVA. Multiple comparison testing was performed using Tukey’s test. When normality was not confirmed, significances were calculated via the Kruskal–Wallis test and multiple comparisons were carried out in this case using Dunn’s multiple comparison test.

## 3. Results

### 3.1. Definition of Reagents

#### Blood-Derived Products

To avoid falsifying the cytokine results, all blood products were initially tested for their cytokine concentration. Blood-derived products were tested for a common panel of cytokines and chemokines (tumor necrosis factor-alpha (TNFα), monocyte chemoattractant protein 1 (MCP1), Interferon-gamma (IFNγ), Interleukin 10 (IL-10), Interleukin 8 (IL-8), Interleukin 6 (IL-6), Interleukin 1ß (IL-1ß), and interleukin-1 receptor antagonist (IL1RA). The results for all tested substances were below or near the control. 

### 3.2. Induction of Senescent State

**Figure 3:**
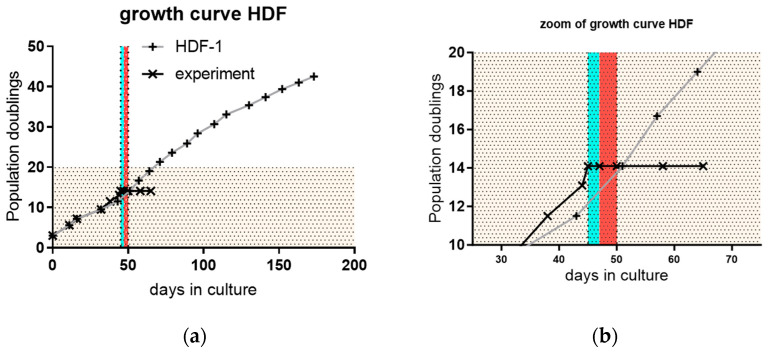

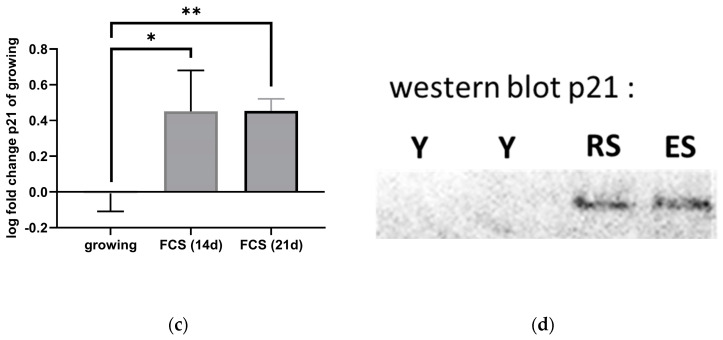
The yellow area represents the range of 0–20 population doublings (PD). The gray line with crosses indicates the continued replicative potential after approximately 40 PD. The black line shows the growth arrest of treated cells for at least 21 days post-treatment. The blue and red lines indicate etoposide and blood product treatment, respectively (**a**). The magnification in (**b**) shows enlarged blue vertical lines. Blue and red display the etoposide treatment time and blood product treatment time for early treatment, respectively. The stopped growth of fibroblasts is indicated by the black line with x, while the untreated cells’ continued growth is shown by the gray line with crosses (**b**). The mRNA-expression of p21 depicted in the column graph shows an increase in p21 mRNA on days 14 d and 21 d (mean of three independent experiments) (**c**). p21 expression on protein level was confirmed in the Western blot by bands at a molecular weight of 21 kD. Two samples of young (Y) cells were analyzed as control, showing no bands at molecular weight 21 kD, while samples of replicative senescent (RS) and etoposide senescent (ES) cells showed a band at this weight (**d**). The statistical significance is indicated by asterisks, where ** represents very significant (*p* < 0.001 to 0.01), * represents significant (*p* < 0.01 to 0.05).

The induction of cellular senescence is a process that is dependent on multiple factors. Therefore, special care was taken to test all the factors necessary to prove that senescence has been established. In all experiments, the cells were used at below 20 population doublings (PD) (yellowish area in Figure 3a PD 0–20), control cells were passaged above PD 40 to show that the replicative potential of cells was not exhausted at the time of the experiments (gray line with crosses). In the zoomed-in section in Figure 3b, the blue vertical line indicates the two days of etoposide treatment and the red vertical line indicates the three days of blood product treatment. The growth arrest of cells is shown by the black line with x. Growth arrest is one major factor of cellular senescence.

Furthermore, the induction of a senescent state was tested by p21-expression on mRNA level, which was increased after 14 and 21 days (FCS-group). Significance was confirmed by simple ANOVA and Tukey´s multiple comparison test with a *p*-value of 0.0127 and 0.0088, respectively (Figure 3c). p21 is a major factor in the development of cellular senescence. Additionally, p16, as another main factor, was tested, as documented in Supplemental Figure S1.

Furthermore, the detection of p21 protein by Western blot was also performed to show that, at the protein level, p21 is also expressed to arrest growth. The blot shows the typical bands at the molecular weight of 21 kD (Figure 3d), confirming the senescent state. Young cells were analyzed twice (Y), compared to a replicative senescent control (RS) and etoposide-induced senescent cells (ES). The faint bands are due to the use of precipitating staining in the absence of any available luminescence device. 

**Figure 4:**
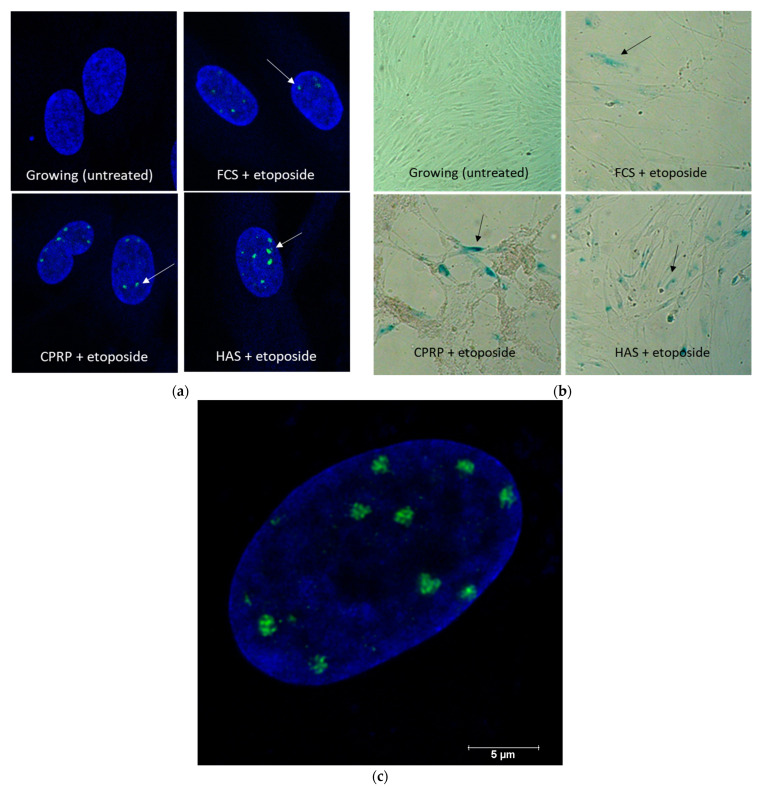
(**a**) γH2AX staining of growing and etoposide-treated HDF. All three blood product treatments (FCS, CPRP, and HAS) show blue DAPI-stained nuclei with green dots (marked by white arrows). These dots indicate DNA lesions stained by immune fluorescence of phosphorylated histone H2AX. The images represent cells on day 4 of early treatment. These experiments were conducted twice (**b**). SA-ß-Gal-stained cells’ blue color indicates the establishment of the senescent phenotype on day 14 of early treatment group. Growing cells did not receive damaging treatment and went on growing to confluence for 14 days. The differences in cell densities between treated and untreated cells are due to the growth arrest induced by etoposide treatment. Experiments were conducted twice. (**c**) Magnification of an etoposide and FCS-treated fibroblast nucleus γH2AX-foci can be clearly seen in green; the scale bar marks the size of the nucleus. The magnification = 65× in (**a**) and 10× in (**b**).

The senescent state was confirmed by γ-H2AXstaining. This staining proved that DNA damage was induced by etoposide treatment. DNA damage and double strand breaks are important inducers of cellular senescence and of SASP. γ-H2AXstaining was positive in all treatments except for the control cells (growing). Staining was performed once. The treatment with etoposide (50 µM) after 2 days of incubation, and subsequent treatment with blood-derived products (FCS, CPRP, and HAS 10% each) for one day, is shown in Figure 4a. γ-H2AX foci (green dots) were located in the nuclei stained with DAPI (blue), demonstrating DNA damage (double-strand breaks) by etoposide treatment. These experiments were conducted with cells at the beginning of their replicative lifespan. SA-ß-Gal staining was positive in all treatments except for the control cells (growing), as shown in Figure 4b. After treatment and lengthy incubation, cells were still vital.

**Figure 5:**
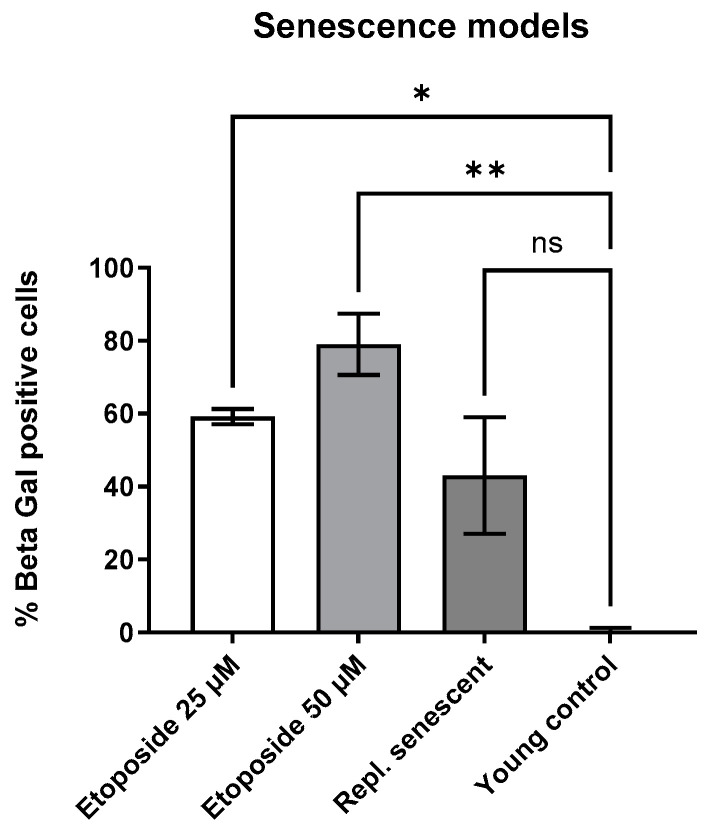
Comparison of different senescence models: 50 µM etoposide leads to a higher percentage of senescent cells. Also, 25 µM replicative senescence models (cells that are passaged close to or until natural growth arrest) show a lower percentage of senescent cells, indicating a not fully senescent state. Experiments were performed in triplicate, except for the 50 µM etoposide treatment, which was analyzed in duplicate. Statistical significance was calculated by Kruskal–Wallis test where ** represents very significant (*p* < 0.001 to 0.01), * represents significant (*p* < 0.01 to 0.05), and ns represents not significant (*p* ≥ 0.05).

Because of the increased residual outgrowth of cells, for further experiments, a higher concentration of etoposide was used (50 µM). This condition leads to a significant increase in SA-beta-gal staining. Figure 5 shows that the percentage of senescent cells compared to young cells was higher in the etoposide treatment groups, with the higher concentration of 50 µM compared to 25 µM for late passage cells (replicative senescent). The percentage was assessed by measuring the total cell count and calculating the percentage of blue-stained cells. This experiment was conducted to show the extent of SA-ß-gal staining. It also shows that the cells did not become 100% senescent.

### 3.3. Proliferation and Outgrowth of HDF Cells

**Figure 6:**
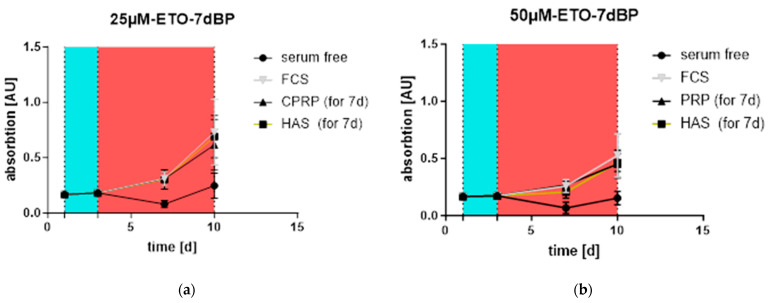
Outgrowth of cells: two independent experiments were conducted in triplicate. (**a**) Treatment with 25 µM etoposide (blue area) led to a decrease in growth but no absolute growth arrest for treatments with FCS (gray with triangles), HAS (cubes with orange line), and CPRP (black with triangles) (concentration: 10%) for seven days (red area) µM. (**b**) Treatment with 50 µM etoposide stopped outgrowth absolutely, indicating a residual growth signal by blood products. Both outgrowth rates did not differ significantly from the FCS control.

The stimulation of “young” HDF cells outgrowth is one of the factors that could lead to the rejuvenation of skin. Young cells overgrow the old ones, regenerating the tissue. Outgrowth was confirmed after the treatment of cells with 25 and 50 µM etoposide, followed by treatment with blood products for 7 days. In Figure 6a,b, the lowest response in the XTT assay can be seen for the negative control and serum-free medium-fed HDFs (shown by the black line with circles). FCS-supplemented cells (gray line with triangles), used as a positive control, showed similar growth to the other blood product treatments: HAS (black box and orange line) and CPRP (black line with triangles). All BPs were applied at a concentration of 10%. There was no significant residual growth of HAS- and CPRP-treated cells compared to the FCS-fed control in the 50 µM etoposide treatment as well as in that with 25 µM.

**Figure 7:**
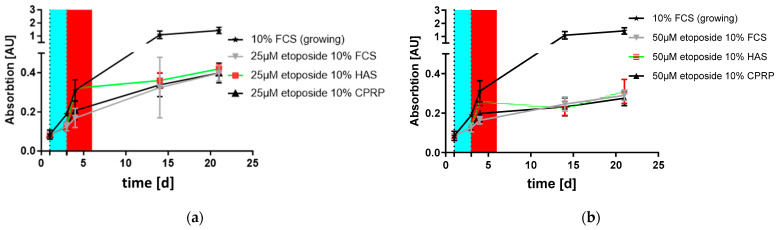
Growth of HDFs without etoposide was assessed in two independent experiments conducted in triplicate. In both figures, 10% FCS (growing) is a condition where no etoposide treatment was applied to track uninhibited cell growth. (**a**) Shows reduced outgrowth by 3 d treatment with blood products (red area) following 2 d etoposide treatment at 25 µM (blue area). (**b**) Shows final treatment conditions of 50 µM etoposide indicating only minor outgrowth, representing establishment of senescent state.

In Figure 7a, it can be observed that the outgrowth rate was relatively high after the 25 µM etoposide treatment compared to that with 50 µM (Figure 7b), indicating a better induction of senescence, while fewer cells could proliferate at the higher concentration of etoposide.

In Supplemental Materials Figure S2, a comparison of different concentrations of HAS and its quick (24 h) anti apoptotic effect post etoposide treatment can be found. This effect contributes to the outgrowth effects of HAS and can also be observed at the 4-day timepoint in Figure 7a,b, which shows an increased response in the HAS group, indicating more cells than in the other groups.

The growth of fibroblasts without damaging treatment and blood product treatment for three days can be found in Supplemental Material Figure S3 indicating an even stronger increase in proliferation compared to the positive control treated with FCS.

### 3.4. Investigations in Cellular Senescence and SASP-Factors IL-6 and IL-8

#### 3.4.1. p21 Expression

The important senescence marker p21 was tested because of the possible senolytic effects of the used blood products. A stable, non-significant p21 expression in all conditions showed no senolytic effect of blood products. The increased p21 expression compared to the growing control is indicative of a stable induction of senescence in all conditions of blood products.

**Figure 8:**
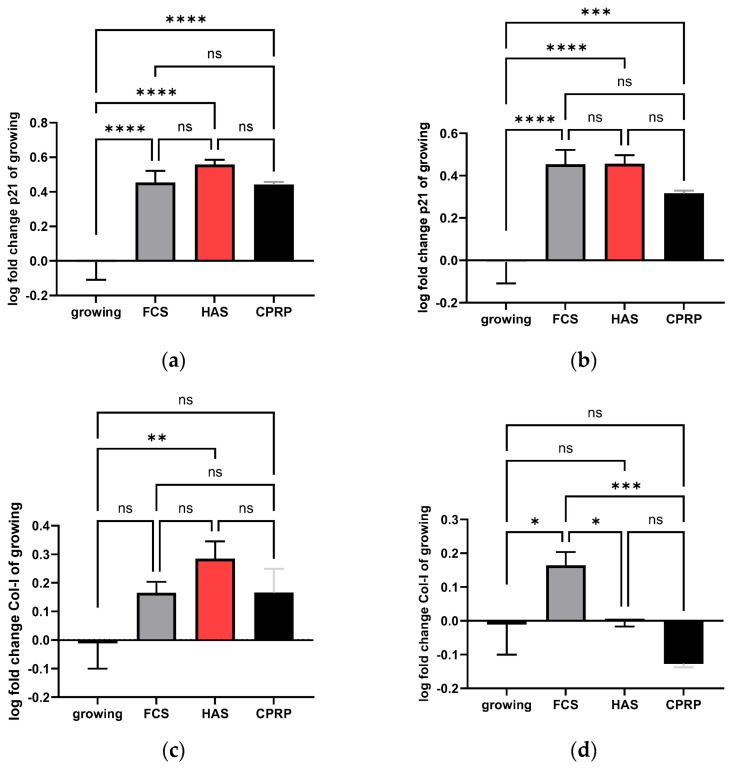
Experiments were performed for three independent treatments and evaluated by qPCR of mRNA expression of p21 for early treatment after 21 d (**a**). Late treatment also shows log fold change values of different timepoints at the final treatment state after 21 d (**b**). Similarly, collagen 1 expression was assessed for early treatment (**c**) and late treatment (**d**) where **** represents significance level below 0.0001, *** represents extremely significant (*p* < 0.0001 to 0.001), ** represents very significant (*p* < 0.001 to 0.01), * represents significant (*p* < 0.01 to 0.05), and ns represents not significant (*p* ≥ 0.05).

p21 expression (Figure 8a) differs significantly from the growing control cells for all treatments. The most prominent increase in p21 expression compared to growing cells was observed for the HAS-treated cells. Nevertheless, this increase was not significant compared to cells treated with FCS and CPRP at the same timepoint.

p21 expression was increased compared to growing cells in all treatments. However, in CPRP-treated cells, p21 expression on day 21 was decreased and less significant compared to growing cells (*p* = 0.0008) (Figure 8b). Data were expressed in logarithmical fold change values and passed Shapiro–Wilk normality testing.

#### 3.4.2. Type I Collagen Expression

Collagen 1 expression can be used as a marker for the general condition of the skin. High values indicate a more vital condition of skin and fibroblasts. Type I collagen (COL1), as a parameter of the extracellular matrix (ECM), was investigated by qPCR. In Figure 8c, an increase in the expression pattern of all three treatments is shown, but only HAS treatment led to statistical significance in the increase in COL1 Expression (*p* = 0.0041) on day 21 in the early treatment group. 

However, at the late stage of treatment (Figure 8d) with blood-derived products, HAS-treated cells showed unaffected growth on day 21. FCS (supplemented normal growth medium) led to a highly significant increase in COL1 expression compared to CPRP-treated cells (*p* = 0.0004), which showed a decreased expression of COL1 compared to growing cells calculated by simple ANOVA with Tukey’s multiple comparison tests. Data were expressed in logarithmical fold change values and passed Shapiro–Wilk normality testing. Interestingly, is the late treatment stage, a totally different pattern than in early treatment was shown.

All experiments were conducted in biological triplicate for qPCR investigations.

The inflammation induced by blood products was determined by IL-6 and IL-8 expression at the protein level. Il-6 and IL-8 are important markers of the SASP and inflammation.

#### 3.4.3. IL-6 Concentration in Supernatant 

**Figure 9:**
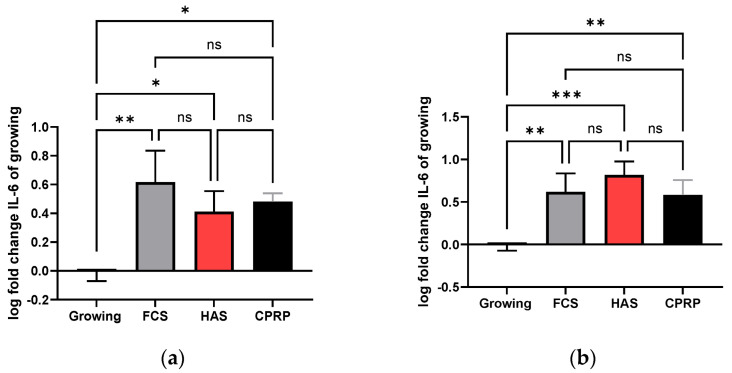

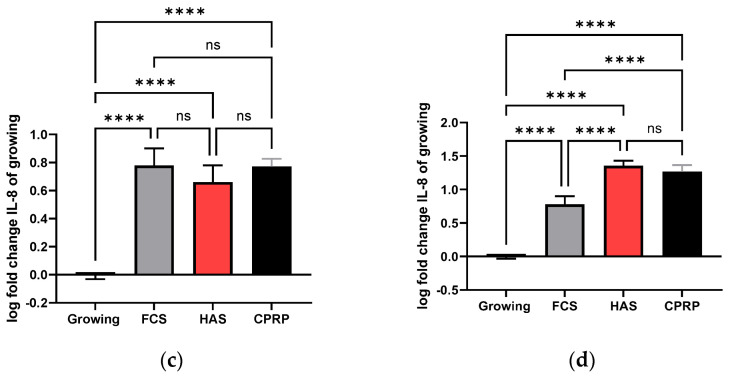
Luminex assay of inflammatory markers. Experiments were performed for three independent treatments. With different BPs, final IL-6 concentration in supernatant normalized to cell count (in all cases) at day 21 (**a**) and late treatment (**b**). The same data are shown for IL-8 concentration after early treatment (**c**) and late treatment (**d**) where **** represents significance level below 0.0001, *** represents extremely significant (*p* < 0.0001 to 0.001), ** represents very significant (*p* < 0.001 to 0.01), * represents significant (*p* < 0.01 to 0.05), and ns represents not significant (*p* ≥ 0.05).

In early treatment on day 21, IL-6 was still increased in all groups compared to the growing control. FCS (normal growth medium) treatment showed the most pronounced increase with a significance of *p* = 0.0019; CPRP and HAS were increased by a significance of 0.0142 and 0.0328, respectively (Figure 9a). In the lengthy treatment, the 21-day timepoint displayed a highly significant increase in IL-6 in all groups, FCS, HAS, and CPRP (*p* = 0.0028, 0.0003, and 0.0042), where HAS increased most significantly (Figure 9b). Data were expressed in logarithmical fold change values; furthermore, they were normalized by the mean cell number of the corresponding experiment. A Shapiro–Wilk normality test indicated normality in all treatments, and simple ANOVA with Tukey’s multiple comparison test was used to calculate significances.

#### 3.4.4. IL-8 Concentration in Supernatant

The IL-8 concentration increased significantly during early treatment for all treatments compared to growing cells on day 21 (*p* < 0.0001). Significance was determined by simple ANOVA with Holm Tukey’s multiple comparison test (Figure 9c). Late treatment (Figure 9d) resulted in differences not only between treatments and growing cells (*p* < 0.0001), but also a significant increase in IL-8 concentration values after HAS and CPRP treatment compared to normal growth medium treatment (FCS) (*p* < 0.0001, significance analyzed by Tukey’s multiple comparison test). Data were expressed in logarithmical fold change values and passed Shapiro–Wilk normality testing. Furthermore, values were normalized by the mean cell number of the corresponding experiment.

Supplemental Material Figure S4 shows the analyses of cell numbers and vitality, confirming growth arrest by etoposide treatment. In Supplemental Material Figure S5, the investigation of cell morphology is documented. Trypsin-treated cells were observed using a Luna cell counter and the size of rounded cells was analyzed. In the early treatment group, only the FCS-treated cells appeared enlarged. During late treatment, an increased cell size was observed for all treatment groups (HAS-, PRP-, and FCS-treated cells).

The time courses for IL-6 and IL-8 concentration measurements are depicted in Supplemental Figure S6.

## 4. Discussion

Autologous blood-derived products, such as platelet-rich plasma and hyperacute serum, show promise for therapeutic use. However, their preparation requires standardization and their biomolecular composition is not sufficiently characterized [35]. Despite these obstacles, ongoing research is making progress in this area [25]. There are still important questions to be answered regarding the timing of treatment, especially considering different cell types, cell states, and other factors [36]. This study specifically focused on the state of cellular senescence of skin HDF cells. The research aims to understand the effects of blood-derived products on cellular senescence, particularly in fibroblasts, and their potential implications for aging and age-related diseases. 

Alessio et al. developed a method for determining the states of cells by using the expression of Ki67, RPS6, and beta-galactosidase. This method allows them to identify healthy cells that enter and leave quiescence through G0-entry, G0, and G0-alert states. They distinguish between quiescence and senescence by examining the expression of RPS6, a marker of active protein synthesis that is present in senescent cells but absent in quiescent cells [37]. In our experiments, we ensured the establishment of a permanent growth arrest by observing markers over a treatment phase of 21 days in total. Despite the growth-promoting signals provided by BP, there was almost no detectable cell growth. 

Cellular senescence is a complex process, and the SA-ß-gal assay is one of the most widely recognized indicators of cellular senescence. However, there are concerns about the reliability of the SA-ß-gal assay, as it can produce false positive results for various reasons, including cell confluence and radical damage to the cells, amongst others. Additionally, SA-ß-gal staining is not easy to quantify. Therefore, SA- ß-gal staining as a single marker cannot confirm the senescent state [38].

Due to a lack of easily quantifiable senescence parameters, cell size was chosen as an easy-to-analyze parameter for morphological changes in senescent cells [39]. Senescent morphology, indicated by the increased size of cells, did not change significantly after CPRP and HAS treatment compared to FCS (normal growth medium). This is consistent with visual observations of cells in SA-ß-gal staining, showing that cells were stained blue but did not change their morphology. This impression was not quantified or tested for statistical significance, but other senescence parameters like p21 and growth arrest confirmed the senescent state of cells. Nevertheless, cell size was consistent with IL-6 and IL-8 expression patterns in late treatment (late pulse with blood-derived products), with HAS and also CPRP treatment especially resulting in increased cell size and production of inflammatory cytokines and chemokines. In contrast, the early treatment caused the opposite effect, with FCS-treated HDF showing more swelling than after HAS and CPRP treatment. This could indicate a dampening effect of BP in early treatment and a stimulating effect in late treatment, which is indicative of an inflammatory (potentially “healing?”) effect. 

The appearance of γH2AX foci was not influenced by 24 h treatment with different blood-derived products representing persistent DNA damage signaling and the development of SASP in all treatments performed. The decision not to count the foci was based on the sole interest in determining the induction of DNA damage. The DNA damage response was further confirmed by the significant upregulation of p21 in all treatments, demonstrating established cellular senescence or at least DNA damage signaling. This upregulation was also validated by the confirmation of increased p21 protein expression through Western blot analysis. Additionally, the upregulation of p16 was confirmed—detailed information can be found in Supplemental Material Figure S1. Furthermore, the confirmation of growth arrest through the tracking of growth curves is emphasized as a crucial aspect in validating cellular senescence. Despite the onset of cellular senescence, the cells were observed to remain viable and metabolically active for 15 days post-treatment.

In summary, we have examined several indicators of cellular senescence [40]: 

A significant upregulation of p21 was observed in all treatments, indicating the activation of this tumor suppressor. However, the late treatment of CPRP showed a less significant increase in p21 expression, which could be an effect of the senolytic activity of this specific BP. 

In terms of SASP, the early treatment of HDF cells with CPRP and HAS caused a decrease in the excretion of IL-6 compared to the FCS-treated positive control, indicating an attenuation of SASP-related inflammatory cytokine levels. The results obtained with the treatment of already senescent cells demonstrated that HAS treatment increased IL-6 and IL-8 excretion significantly. In the case of IL-8, the increase in secretion in HAS- and CPRP-treated cells was significantly higher than after FCS treatment. An interesting topic for further investigation is the question whether the increased cytokine release leads to the attraction of immune cells and clearance of senescent cells in vivo. 

The growth of HDF was strongly promoted by HAS and even more by CPRP, and hints at the additional quick anti-apoptotic effect of HAS after damaging treatment were observed. 

The use of FCS supplementation as a control is questionable considering the origin of the serum. FCS contains a mixture of growth factors and therefore may not constitute an ideal control for experiments with other blood-derived products, as it could mask some of their effects or make them seem less pronounced than they would be under normal physiological conditions. The wound-healing process is a complex multistep process in which senescence plays a role in both positive and negative ways. 

Senescent cells accumulate in old skin, leading to suboptimal wound healing, especially in old organisms or people with diabetes. The role of cellular senescence in wound healing is still not fully understood, and further research is needed to clarify its contribution to normal and pathological wound healing [41]. 

Regarding type I collagen, the expression pattern in early and late treatment groups is totally different. This might be related to different growth factor contents in the different blood products. Studies have shown that certain signals, such as transforming growth factor beta 1 (TGF-β1) and epidermal growth factor (EGF), increase Col1a1 expression [42]. In our study, it seems that these effects play a bigger role in the onset of senescence (early treatment). It is possible that these signals are more active during senescence and thus contribute to the increase in Col1a1 expression. Additionally, CD40L might play a role in the different reactions to HAS, CPRP, and FCS treatments. The differences between these blood products were investigated by Kardos et al. [25]. Further research has to be conducted to shed light on this interesting topic.

Furthermore, the increased secretion of IL-,8, as confirmed by Luminex assay in the late treatment group, could also influence collagen 1 expression in an autocrine manner. IL-8 itself affects the morphology and possibly the function of fibroblasts in collagen lattices, leading to alterations such as disrupted microfilament organization and a change in cell shape [43]. These morphological changes may indirectly influence collagen synthesis. There appears to be a complex relationship between IL-8 and collagen 1. IL-8 also appears to downregulate collagen synthesis in skin fibroblasts. One study found that collagen type I inhibited IL-8 secretion from human neutrophils and downregulated IL-8 mRNA [44]. Regarding the results in our study, the upregulation of IL-8 could have downregulated Collagen 1 and vice versa. This downregulation of Collagen 1 and the additional expression of metalloproteases could make the tissue more accessible for immune cells [45]. Also, the proliferation of undamaged fibroblast cells could be induced. While collagen expression is increased in fibroblast cells in early treatment, the decreased collagen 1 in the late treatment group could point in the direction of tissue remodeling in aged skin.

Moreover, the induction of type I collagen production, especially by HAS (in early treatment), also contributes to the alleviation of the negative effects caused by etoposide treatment. Summing up these observations, HAS and CPRP could have beneficial effects for skin rejuvenation by influencing dermal HDFs with regard to senescence (including the induction of the proliferation of HDF cells).

## 5. Conclusions

In general, the rejuvenation of skin by blood products could be confirmed by the increased outgrowth of cells with residual growing potential. Treatments with HAS and CPRP showed increased cell growth compared to the positive control (FCS). The inflammatory cytokine excretion (IL-6 and IL-8) of senescent fibroblasts could be shown to be altered, with HAS and CPRP demonstrating similar effects to the positive control FCS treatment. Notably, in the late treatment group, IL-8 secretion was significantly increased by CPRP and HAS treatment, even compared to the positive control (FCS). This suggests that the inflammatory signal can be heightened in senescent cells, potentially attracting immune cells for senescent cell clearance among other functions. Additionally, the down-regulation of Collagen 1 and tissue remodeling could be caused by increased IL-8 expression. This study emphasizes the importance of the timing of treatment and the cellular state of skin cells, highlighting their significance in skin aging and healing. The findings also suggest that applying the conditions that were investigated in this study to skin models could better mimic natural skin conditions. Finally, investigating the interplay with immune cells is deemed to be of great importance. To our knowledge, we were the first to investigate this kind of fibroblast cell state (fibroblasts in the development of senescence and already senescent cells) in one study. In any case, the combined effects, like those we have shown in this study, may be considered and treatment outcomes will be dependent on the exact composition of the respective skin of the patients.

## Data Availability

Data are contained within the article and Supplementary Materials.

## Supplementary Materials

The following supporting information can be downloaded at https://www.mdpi.com/article/10.3390/cimb46030122/s1, Section S1: p16 mRNA Expression; Section S2: XTT Assay and Caspase 3/7 Analysis; Section S3: Growth Without Damaging Treatment; Section S4: Cell Numbers; Section S5: Cell Morphology; Section S6: Time Course of IL-6 and IL-8 Analysis; Section S7: Western Blot Raw Data; Section S8: Data of Cell Line; Figure S1: p16 expression of etoposide-treated HDF; Figure S2: Different HAS condensations. Two independent experiments were performed in triplicates (**a**) 25 µM (**b**) 50 µM etoposide (48 h) and 24 h of BP treatment showing increased growth of HAS treat-ed cells correlated with decreased CASP3/7 activity indicating prevention of apoptosis by HAS at the same timepoint with 25 (**c**) and 50 µM (**d**) etoposide treated HDFs. Where **** represents significance level below 0.0001, *** represents extremely significant (*p* < 0.0001 to 0.001), ** represents very significant (*p* < 0.001 to 0.01), * represents significant (*p* < 0.01 to 0.05), and ns represents not significant (*p* ≥ 0.05); Figure S3: Without damaging treatment (**a**) but a 3 day pulse HAS and CPRP both show a higher growth than FCS 10%. When HAS is diluted to 1% and 5% the growth is decreased compared to 10% HAS but still similar to FCS 10% (**b**); Figure S4: Three independent experiments. Early treatment, the number of living cells stayed nearly the same in all three treatment groups (**a**). Continuously growing (dotted line with stars) cells reach confluency after 4–5 days after seeding. (**b**) Vitaliy was measured by trypan exclusion and showed less vitality in Blood product treatment (HAS and CPRP), when finally harvested at day 21. Late treatment (**c**) cell numbers decreased in CPRP treatment and even more in HAS treatment (**d**) vitalities showed a significant decrease in CPRP and HAS compared to both control conditions (growing and FCS). Where **** represents significance level below 0.0001, ** represents very significant (*p* < 0.001 to 0.01), * represents significant (*p* < 0.01 to 0.05), and ns represents not significant (*p* ≥ 0.05); Figure S5: Cell morphology was determined by cell size and was not affected significantly in early treatment setup three independent experiments were performed (**a**) in HAS and CPRP treated HDF cells. The size of FCS (normal growth medium) treated cells increased significantly with *p* = 0.0028. Contrary, late treatment setup (**b**) resulted in a highly significant increase in the size of HAS treated cells (*p* < 0.0001) and less but also significant increase in CPRP group (*p* = 0.0093). Significances were calculated by Kruskal Wallis test, data that did not pass the normality test (Shapiro–Wilk), a multiple comparison by Dunn’s multiple comparison test was performed (*n* = 3 or 4). Column graphs represent the 21-day timepoint at the end of the experiment. Where **** represents significance level below 0.0001, ** represents very significant (*p* < 0.001 to 0.01), and ns represents not significant (*p* ≥ 0.05); Figure S6: Time course of treatments which were done in three independent experiments. (**a**,**b**) IL-6 early and late respectively and IL-8 early (**c**) and late (**d**) as well as p21 early (**e**) and late treatment (**f**) Collagen 1 time course can be seen for early treatment in (**g**) and late treatment in (**h**); Figure S7: Original image of the Western bolt; Figure S8: Information about the cell line. Reference [46] is cited in the Supplementary Materials.

## References

[1] Baker D.J., Wijshake T., Tchkonia T., LeBrasseur N.K., Childs B.G., van de Sluis B., Kirkland J.L., van Deursen J.M. (2011). Clearance of p16Ink4apositive senescent cells delays ageingassociated disorders. Nature.

[2] Baker D.J., Childs B.G., Durik M., Wijers M.E., Sieben C.J., Zhong J., Saltness R.A., Jeganathan K.B., Verzosa G.C., Pezeshki A. (2016). Naturally occurring p16(Ink4a)-positive cells shorten healthy lifespan. Nature.

[3] Hayflick L., Moorhead P.S. (1961). The serial cultivation of human diploid cell strains. Exp. Cell Res..

[4] Toussaint O., Dumont P., Remacle J., Dierick J.-F., Pascal T., Frippiat C., Magalhaes J.P., Zdanov S., Chainiaux F. (2002). Stress-induced premature senescence or stress-induced senescence-like phenotype: One in vivo reality, two possible definitions?. Sci. World J..

[5] van Deursen J.M. (2014). The role of senescent cells in ageing. Nature.

[6] Childs B.G., Durik M., Baker D.J., van Deursen J.M. (2015). Cellular senescence in aging and age-related disease: From mechanisms to therapy. Nat. Med..

[7] Velarde M.C., Demaria M., Campisi J. (2013). Senescent cells and their secretory phenotype as targets for cancer therapy. Cancer Aging Bench Clin..

[8] Krizhanovsky V., Yon M., Dickins R.A., Hearn S., Simon J., Miething C., Yee H., Zender L., Lowe S.W. (2008). Senescence of Activated Stellate Cells Limits Liver Fibrosis. Cell.

[9] Rea I.M., Gibson D.S., McGilligan V., McNerlan S.E., Alexander H.D., Ross O.A. (2018). Age and Age-Related Diseases: Role of Inflammation Triggers and Cytokines. Front. Immunol..

[10] Rodier F., Coppé J.-P., Patil C.K., Hoeijmakers W.A.M., Muñoz D.P., Raza S.R., Freund A., Campeau E., Davalos A.R., Campisi J. (2009). Persistent DNA damage signalling triggers senescence-associated inflammatory cytokine secretion. Nat. Cell Biol..

[11] Marin I., Serrano M., Pietrocola F. (2023). Recent insights into the crosstalk between senescent cells and CD8 T lymphocytes. Npj Aging.

[12] Loughery J., Cox M., Smith L.M., Meek D.W. (2014). Critical role for p53-serine 15 phosphorylation in stimulating transactivation at p53-responsive promoters. Nucleic Acids Res..

[13] Rodier F., Kim S.-H., Nijjar T., Yaswen P., Campisi J. (2005). Cancer and aging: The importance of telomeres in genome maintenance. Int. J. Biochem. Cell Biol..

[14] Baldwin E.L., Osheroff N. (2005). Etoposide, topoisomerase II and cancer. Curr. Med. Chem. Anticancer. Agents.

[15] Petrova N.V., Velichko A.K., Razin S.V., Kantidze O.L. (2016). Small molecule compounds that induce cellular senescence. Aging Cell.

[16] Probin V., Wang Y., Bai A., Zhou D. (2006). Busulfan selectively induces cellular senescence but not apoptosis in WI38 fibroblasts via a p53-independent but extracellular signal-regulated kinase-p38 mitogen-activated protein kinase-dependent mechanism. J. Pharmacol. Exp. Ther..

[17] Odeh A., Dronina M., Domankevich V., Shams I., Manov I. (2020). Downregulation of the inflammatory network in senescent fibroblasts and aging tissues of the long-lived and cancer-resistant subterranean wild rodent, Spalax. Aging Cell.

[18] Griffiths T.W., Watson R.E.B., Langton A.K. (2023). Skin ageing and topical rejuvenation strategies. Br. J. Dermatol..

[19] Farage M.A., Miller K.W., Elsner P., Maibach H.I. (2013). Characteristics of the Aging Skin. Adv. Wound Care.

[20] Boukamp P. (2005). Skin aging: A role for telomerase and telomere dynamics?. Curr. Mol. Med..

[21] Waldera Lupa D.M., Kalfalah F., Safferling K., Boukamp P., Poschmann G., Volpi E., Götz-Rösch C., Bernerd F., Haag L., Huebenthal U. (2015). Characterization of Skin Aging-Associated Secreted Proteins (SAASP) Produced by Dermal Fibroblasts Isolated from Intrinsically Aged Human Skin. J. Investig. Dermatol..

[22] Ghosh K., Capell B.C. (2016). The Senescence-Associated Secretory Phenotype: Critical Effector in Skin Cancer and Aging. J. Investig. Dermatol..

[23] Charles-de-Sá L., Gontijo-de-Amorim N.F., Takiya C.M., Borojevic R., Benati D., Bernardi P., Sbarbati A., Rigotti G. (2018). Effect of Use of Platelet-Rich Plasma (PRP) in Skin with Intrinsic Aging Process. Aesthet. Surg. J..

[24] Maisel-Campbell A.L., Ismail A., Reynolds K.A., Poon E., Serrano L., Grushchak S., Farid C., West D.P., Alam M. (2019). A systematic review of the safety and effectiveness of platelet-rich plasma (PRP) for skin aging. Arch. Dermatol. Res..

[25] Kardos D., Simon M., Vácz G., Hinsenkamp A., Holczer T., Cseh D., Sárközi A., Szenthe K., Bánáti F., Szathmary S. (2019). The Composition of Hyperacute Serum and Platelet-Rich Plasma Is Markedly Different despite the Similar Production Method. Int. J. Mol. Sci..

[26] Jia C., Lu Y., Bi B., Chen L., Yang Q., Yang P., Guo Y., Zhu J., Zhu N., Liu T. (2017). Platelet-rich plasma ameliorates senescence-like phenotypes in a cellular photoaging model. RSC Adv..

[27] Kuten O., Simon M., Hornyák I., de Luna-Preitschopf A., Nehrer S., Lacza Z. (2018). The Effects of Hyperacute Serum on Adipogenesis and Cell Proliferation of Mesenchymal Stromal Cells. Tissue Eng. Part A.

[28] Jeyakumar V., Niculescu-Morzsa E., Bauer C., Lacza Z., Nehrer S. (2017). Platelet-Rich Plasma Supports Proliferation and Redifferentiation of Chondrocytes during In Vitro Expansion. Front. Bioeng. Biotechnol..

[29] Fabi S., Sundaram H. (2014). The potential of topical and injectable growth factors and cytokines for skin rejuvenation. Facial Plast. Surg..

[30] Passaretti F., Tia M., D’Esposito V., de Pascale M., Del Corso M., Sepulveres R., Liguoro D., Valentino R., Beguinot F., Formisano P. (2014). Growth-promoting action and growth factor release by different platelet derivatives. Platelets.

[31] Lammermann I., Terlecki-Zaniewicz L., Weinmullner R., Schosserer M., Dellago H., de Matos Branco A.D., Autheried D., Sevcnikar B., Kleissl L., Berlin I. (2018). Blocking negative effects of senescence in human skin fibroblasts with a plant extract. NPJ Aging Mech. Dis..

[32] Neubauer M., Kuten O., Stotter C., Kramer K., de Luna A., Muellner T., Lacza Z., Nehrer S. (2019). The Effect of Blood-Derived Products on the Chondrogenic and Osteogenic Differentiation Potential of Adipose-Derived Mesenchymal Stem Cells Originated from Three Different Locations. Stem Cells Int..

[33] Otahal A., Kuten-Pella O., Kramer K., Neubauer M., Lacza Z., Nehrer S., de Luna A. (2021). Functional repertoire of EV-associated miRNA profiles after lipoprotein depletion via ultracentrifugation and size exclusion chromatography from autologous blood products. Sci. Rep..

[34] Dimri G.P., Lee X., Basile G., Acosta M., Scott G., Roskelley C., Medrano E.E., Linskens M., Rubelj I., Pereira-Smith O. (1995). A biomarker that identifies senescent human cells in culture and in aging skin in vivo. Proc. Natl. Acad. Sci. USA.

[35] Chahla J., Cinque M.E., Piuzzi N.S., Mannava S., Geeslin A.G., Murray I.R., Dornan G.J., Muschler G.F., LaPrade R.F. (2017). A Call for Standardization in Platelet-Rich Plasma Preparation Protocols and Composition Reporting: A Systematic Review of the Clinical Orthopaedic Literature. J. Bone Jt. Surg. Am..

[36] Martínez C.E., Smith P.C., Palma Alvarado V.A. (2015). The influence of platelet-derived products on angiogenesis and tissue repair: A concise update. Front. Physiol..

[37] Alessio N., Aprile D., Cappabianca S., Peluso G., Di Bernardo G., Galderisi U. (2021). Different Stages of Quiescence, Senescence, and Cell Stress Identified by Molecular Algorithm Based on the Expression of Ki67, RPS6, and Beta-Galactosidase Activity. Int. J. Mol. Sci..

[38] Soubeyrand S., Pope L., Haché R.J.G. (2010). Topoisomerase IIalpha-dependent induction of a persistent DNA damage response in response to transient etoposide exposure. Mol. Oncol..

[39] Biran A., Zada L., Abou Karam P., Vadai E., Roitman L., Ovadya Y., Porat Z., Krizhanovsky V. (2017). Quantitative identification of senescent cells in aging and disease. Aging Cell.

[40] Hernandez-Segura A., Nehme J., Demaria M. (2018). Hallmarks of Cellular Senescence. Trends Cell Biol..

[41] Wyld L., Bellantuono I., Tchkonia T., Morgan J., Turner O., Foss F., George J., Danson S., Kirkland J.L. (2020). Senescence and Cancer: A Review of Clinical Implications of Senescence and Senotherapies. Cancers.

[42] Liguori T.T.A., Liguori G.R., Moreira L.F.P., Harmsen M.C. (2018). Fibroblast growth factor-2, but not the adipose tissue-derived stromal cells secretome, inhibits TGF-β1-induced differentiation of human cardiac fibroblasts into myofibroblasts. Sci. Rep..

[43] Iocono J.A., Colleran K.R., Remick D.G., Gillespie B.W., Ehrlich H.P., Garner W.L. (2000). Interleukin-8 levels and activity in delayed-healing human thermal wounds. Wound Repair. Regen..

[44] Quan W.-Y., Ko J.-A., Yanai R., Nakamura Y., Nishida T. (2010). Integrin-mediated inhibition of interleukin-8 secretion from human neutrophils by collagen type I. J. Leukoc. Biol..

[45] PubMed Central Can Platelet-Rich Plasma Be Used for Skin Rejuvenation? Evaluation of Effects of Platelet-rich Plasma on Human Dermal Fibroblast. https://www.ncbi.nlm.nih.gov/pmc/articles/PMC3229934/.

[46] Hernandez-Segura A., Rubingh R., Demaria M. (2019). Identification of stable senescence-associated reference genes. Aging Cell.

