# Supplementary material for: Effects of Blood-Derived Products on Cellular Senescence and Inflammatory Response: A Study on Skin Rejuvenation
Source: Curr Issues Mol Biol. 2024 Feb 28;46(3):1865–85. doi: 10.3390/cimb46030122 (PMC10968991; doi:10.3390/cimb46030122)
Supplement: Supplementary file 1 [file cimb-46-00122-s001.zip › cimb-2846202-supplementary.pdf]

Supplementary Materials

S1. p16 mRNA Expression

Method:

Analysis of p16: RNA was extracted by Qiagen RNeasy Kit and then RNA was reverse transcribed with iScript Reverse Transcriptase (Bio-Rad Laboratories, Hercules, CA, USA) following the protocols of supplier. Analysis of p16 was performed with iTaq Universal SYBR Green Supermix (Bio-Rad Laboratories) in a reaction total volume of 10 µL with forward primer for p16: 5′-CAACGCACCGAATAGTTACG-3′ and reverse Primer 5′-AC-CAGCGTGTCCAGGAAG-3′ fold change was calculated by delta delta Ct method with reference genes VAMP7 and TUBA1A according to Alejandra Hernandez-Segura et. al [1].

Result:

Supplemental Figure S1 p16 as an additional senescence marker was evaluated showing increased expression after etoposide treatment with a peak expression after 4 d after etoposide exposure of about 3-fold and after 15 d a second peak at about 4.5 fold expression.

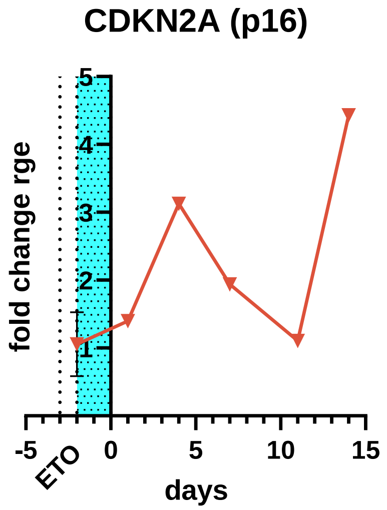

Figure S1. p16 expression of etoposide-treated HDF.

S2. XTT Assay and Caspase 3/7 Analysis

Method: Apo-ONE® Caspase-3/7 assay

Cells were treated the same way as for the XTT assay and assay procedure of Apo-ONE® Caspase-3/7 (Promega, Madison, WI, USA) assay according to manufacturer's instructions, Briefly, 100 µL of Apo-ONE® Caspase-3/7 Reagent were added to each well of a black 96-well plate containing 100 µL of blank, control or cells in culture. Contents of wells was gently mixed using a plate shaker at 300–500 rpm from 30 seconds up to read time Plates were covered and incubated for 4 hours. fluorescence was measured at 499 nm with an emission maximum at a wavelength of 521 nm (PowerWave microplate spectrophotometer (BioTek, Winooski, VT, USA)).

Result:

Comparing different concentrations of HAS (1%, 5% and 10%) to CPRP and FCS both 10%, treated with 25 µM etoposide (Supplemental Figure S2a) and 50 µM etoposide (Supplemental Figure S2b) after 24h of regeneration after etoposide pulse (48 h) shows a significantly higher absorption of 5% and 10% treatment and dose dependent increase in growth (indicated by higher absorption in XTT assay) which is less pronounced in 50 µM etoposide treated cells but still significant by one way ANOVA with Tukey's multiple comparisons test. This increase in growth is well correlated with a decrease in CASP 3/7 activity shown in Supplemental Figure 2c, d showing apoptosis inhibition by HAS after 24 h post etoposide treatment.

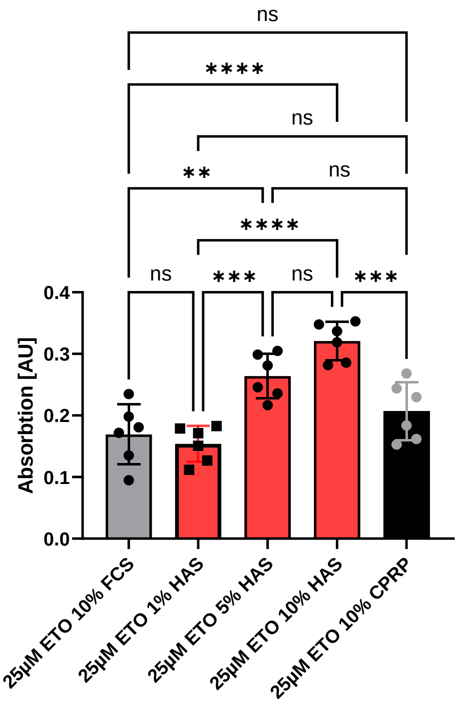

(**a**)

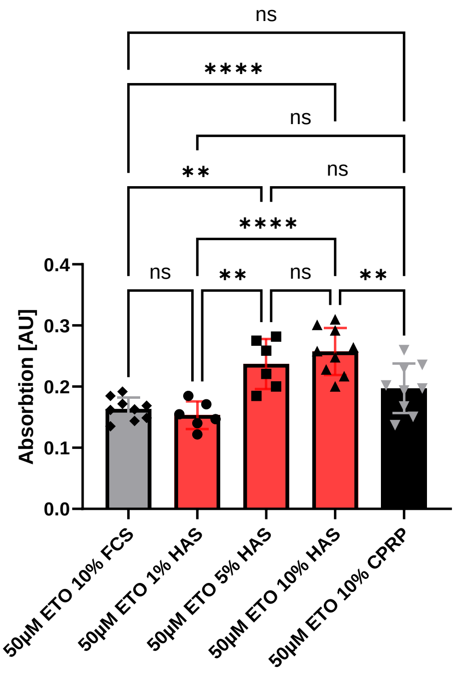

(**b**)

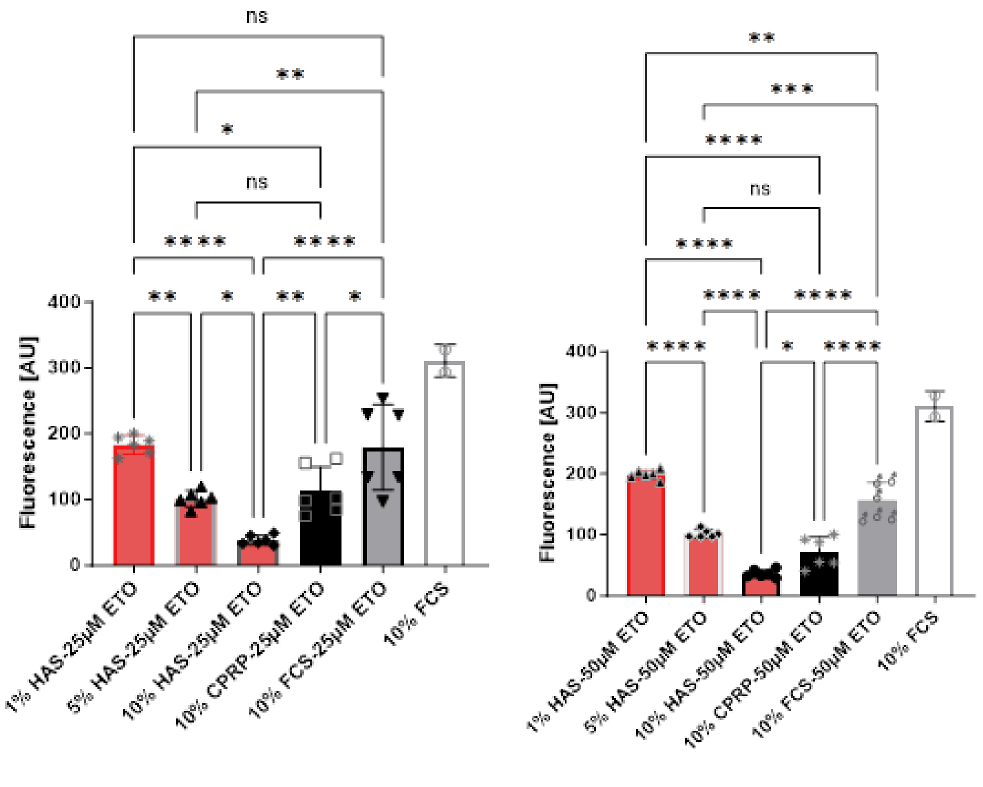

(**c**) (**d**)

Figure S2. Different HAS condensations. Two independent experiments were performed in triplicates (**a**) 25 µM (**b**) 50 µM etoposide (48 h) and 24 h of BP treatment showing increased growth of HAS treated cells correlated with decreased CASP3/7 activity indicating prevention of apoptosis by HAS at the same timepoint with 25 (**c**) and 50 µM (**d**) etoposide treated HDFs. Where **** represents significance level below 0.0001, *** represents extremely significant ($p < 0.0001$ to 0.001), ** represents very significant ($p < 0.001$ to 0.01), * represents significant ($p < 0.01$ to 0.05), and ns represents not significant ($p \geq 0.05$).

S3. Growth Without Damaging Treatment

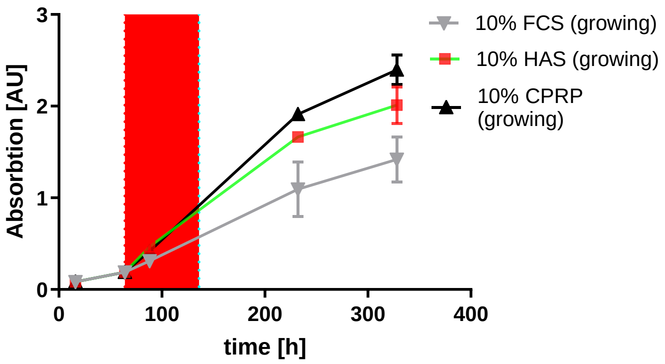

(a)

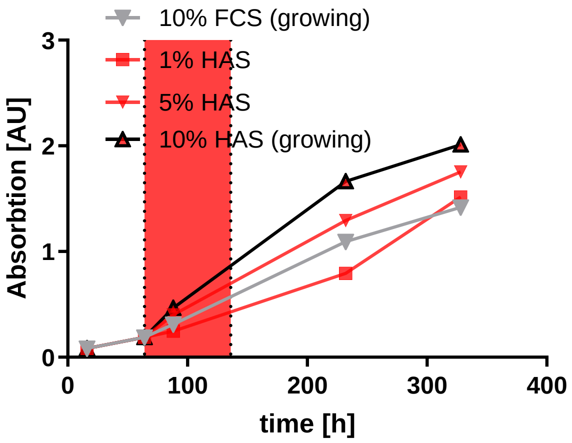

(b)

Figure S3. Without damaging treatment (**a**) but a 3 day pulse HAS and CPRP both show a higher growth than FCS 10%. When HAS is diluted to 1% and 5% the growth is decreased compared to 10% HAS but still similar to FCS 10% (**b**).

S4. Cell Numbers

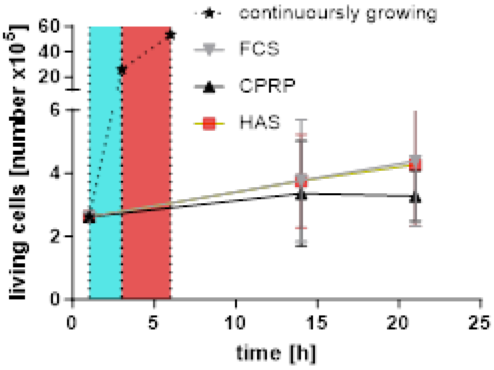

(**a**)

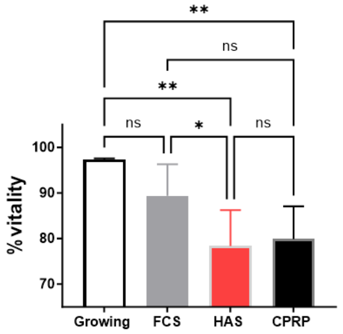

(**b**)

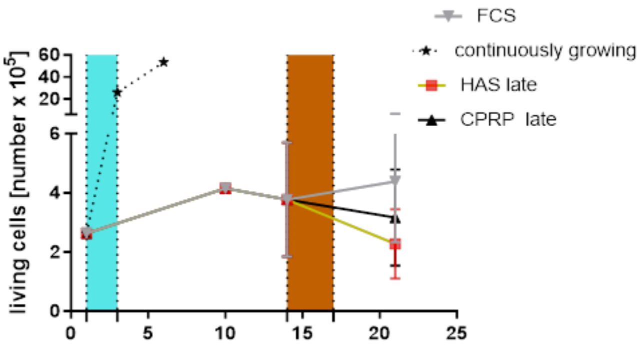

(**c**)

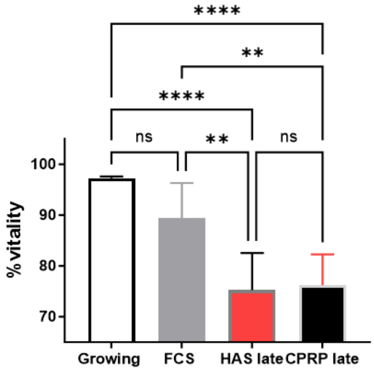

(**d**)

Figure S4. Three independent experiments. Early treatment, the number of living cells stayed nearly the same in all three treatment groups (**a**). Continuously growing (dotted line with stars) cells reach confluency after 4–5 days after seeding. (**b**) Vitaliy was measured by trypan exclusion and showed less vitality in Blood product treatment (HAS and CPRP), when finally harvested at day 21. Late treatment (**c**) cell numbers decreased in CPRP treatment and even more in HAS treatment (**d**) vitalities showed a significant decrease in CPRP and HAS compared to both control conditions (growing and FCS). Where **** represents significance level below 0.0001, ** represents very significant (p < 0.001 to 0.01), * represents significant (p < 0.01 to 0.05), and ns represents not significant ($p \geq 0.05$).

S5. Cell Morphology

Cell size was determined after trypsinization with Luna cell counter, all cells were rounded and well comparable.

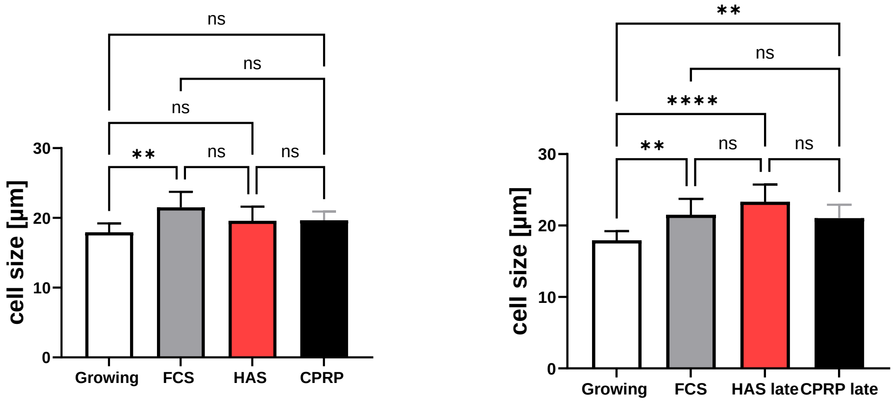

(**a**) (**b**)

Figure S5. Cell morphology was determined by cell size and was not affected significantly in early treatment setup three independent experiments were performed (**a**) in HAS and CPRP treated HDF cells. The size of FCS (normal growth medium) treated cells increased significantly with $p = 0.0028$. Contrary, late treatment setup (**b**) resulted in a highly significant increase in the size of HAS treated cells ($p < 0.0001$) and less but also significant increase in CPRP group ($p = 0.0093$). Significances were calculated by Kruskal Wallis test, data that did not pass the normality test (Shapiro–Wilk), a multiple comparison by Dunn's multiple comparison test was performed ($n = 3$ or 4). Column graphs represent the 21-day timepoint at the end of the experiment. Where **** represents significance level below 0.0001, ** represents very significant ($p < 0.001$ to 0.01), and ns represents not significant ($p \geq 0.05$).

S6. Time Course of IL-6 and IL-8 Analysis

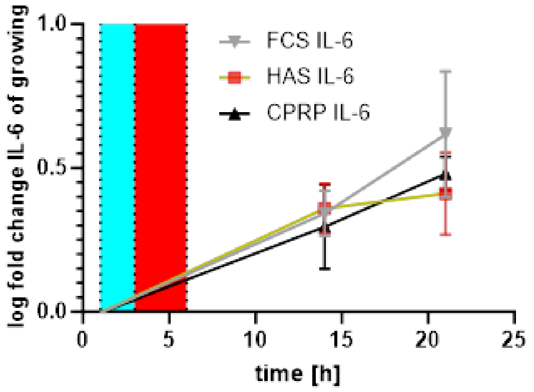

(**a**)

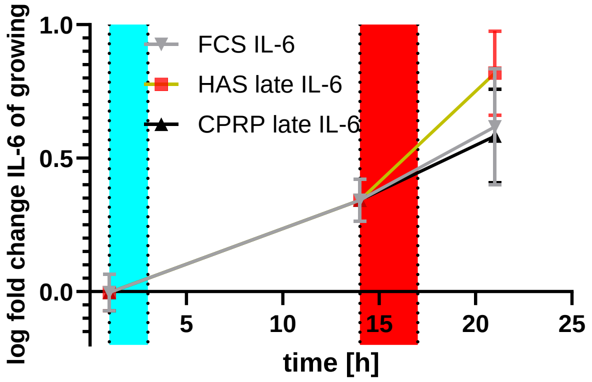

(**b**)

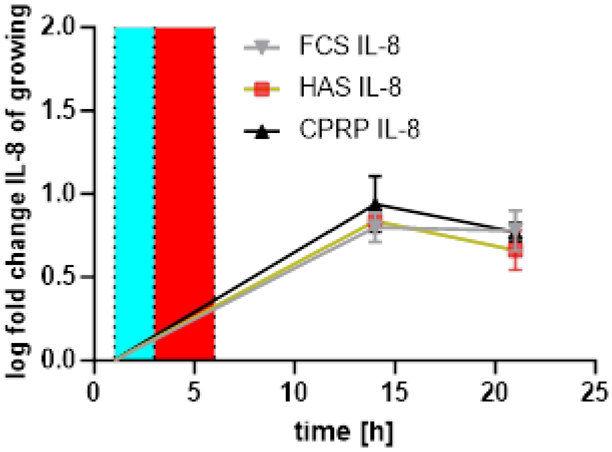

(**c**)

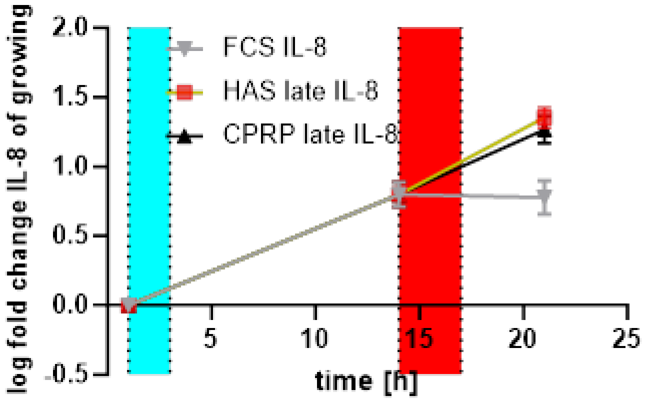

(**d**)

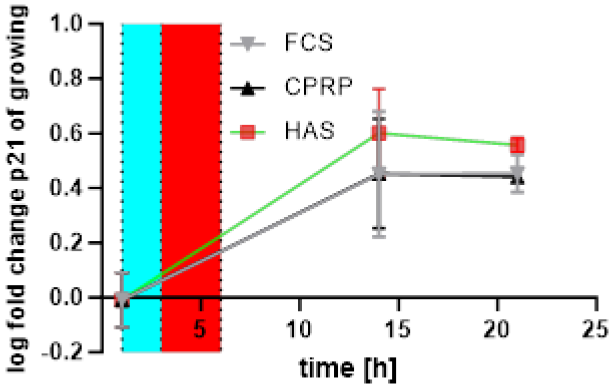

(**e**)

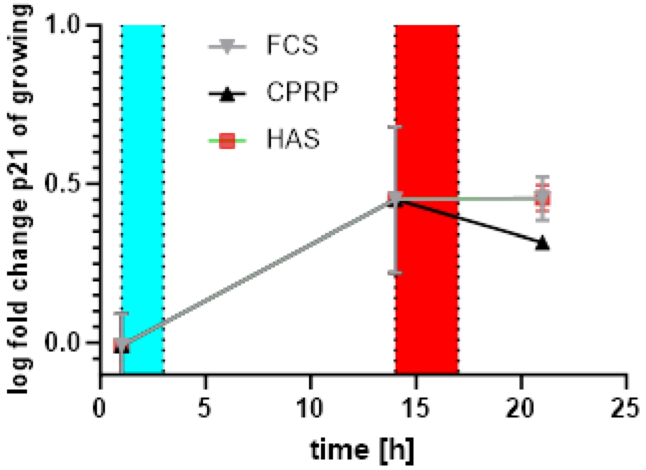

(**f**)

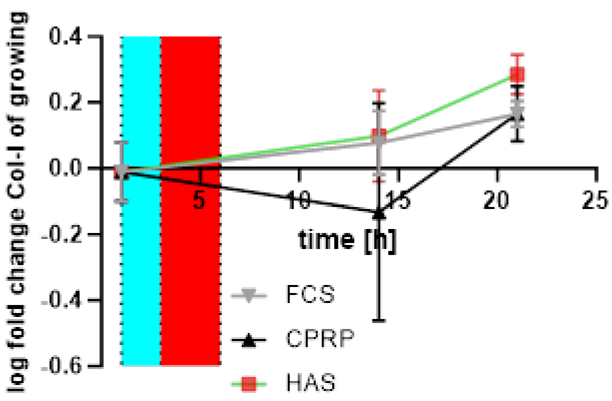

(**g**)

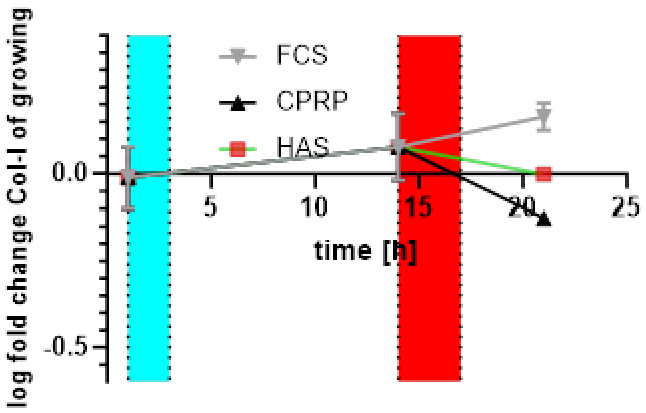

(**h**)

Figure S6. Time course of treatments which were done in three independent experiments. (**a**,**b**) IL-6 early and late respectively and IL-8 early (**c**) and late (**d**) as well as p21 early (**e**) and late treatment (**f**) Collagen 1 time course can be seen for early treatment in (**g**) and late treatment in (**h**).

S7. Western Blot Raw Data

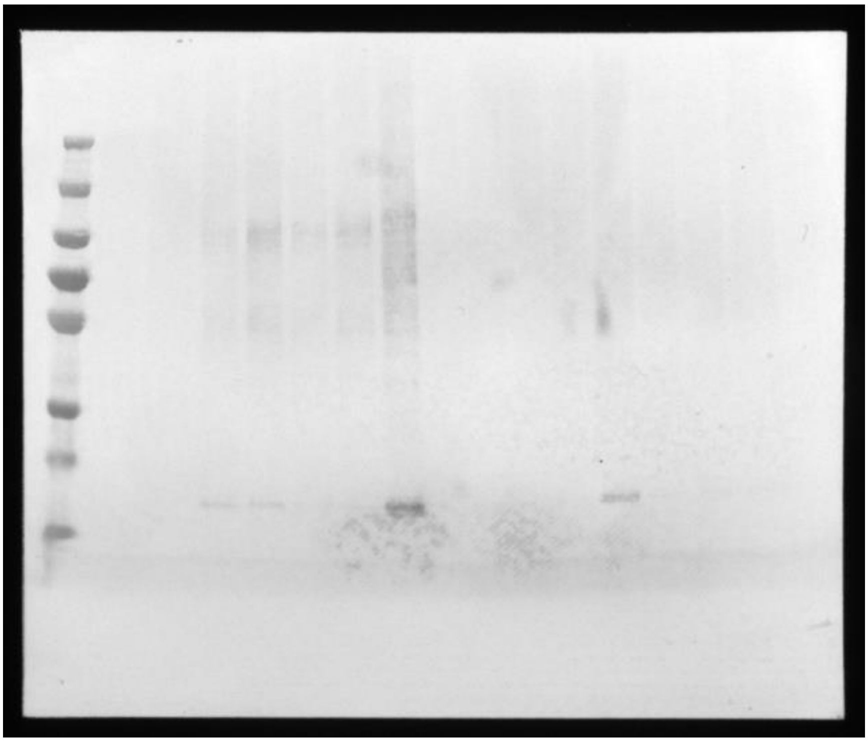

Figure S7. Original image of the Western bolt.

Western blot was done with a precipitating method, what is the reason for faint p21 bands. The first 4 lanes next to the mol weight standard are the ones in the paper.

S8. Data of Cell Line

CellResearchCorp

tebu-bio

CellResearch Corporation Pte Ltd
#04-01F Clinical Research Centre
Block MD-11, 10 Medical Drive
Singapore 117597
Tel: (+65) 6874-3357 Email: info@cellresearchcorp.com

www.tebu-bio.com

CERTIFICATE OF ANALYSIS

**Product code:**	NF170
**Product Description:**	Fibroblasts isolated from normal skin, cryopreserved

**Donor Screen Information:**	**Results**
Age	51 years old
Sex	Male
Race	Caucasian
Location of tissue	Rt Wrist Skin
Primary cell culture method	Explant technique
Cell culture medium	DMEM/10% FCS
Cell passage	3
Date of cryopreservation	
Cell number (cells/vial)	>500,000

**Virus Testing:**	
HIV-1 PCR Test	Negative
HBV DNA PCR Test	Negative
HCV RNA PCR Test	Negative

Figure S8. Information about the cell line.

Reference

1. Hernandez-Segura, A.; Rubingh, R.; Demaria, M. Identification of stable senescence-associated reference genes. *Aging Cell* **2019**, *18*, e12911. https://doi.org/10.1111/acel.12911.
